# Supplementary material for: The prevalence of hyperglycemia and its impact on mortality among people living with HIV in Georgia
Source: PLoS One. 2022 Oct 27;17(10):e0276749. doi: 10.1371/journal.pone.0276749 (PMC9612544; doi:10.1371/journal.pone.0276749)
Supplement: Attachment — Submitted filename: Response to Reviewers.docx [file pone.0276749.s002.docx]

**A Rebuttal Letter**

Dear Dr. Alana T Brennan

We appreciate the opportunity to revise our manuscript for further review at Plos One. The manuscript has been substantially revised in accordance with comments provided by Editorial Board and reviewers. The manuscript has been formatted by the instruction provided. Below are point-by-point responses to the Academic Editor and reviewers’ comments:

**Reviewer #1 (**Emma M. Kileel)**:** Increasing prevalence of noncommunicable diseases among people living with HIV is of growing concern globally. The authors aim to characterize the presence of hyperglycemia and its associated risk of mortality among a large cohort of HIV positive individuals in Georgia. Overall, these results fill an important knowledge gap in the current literature and should be published. However, authors should address or take into consideration the comments below:

Comment: In the introduction, the authors clearly establish an association between specific classes of ART regimens - including PIs, NRTIs, and some NNRTIs - and ART duration with type 2 diabetes. Do the authors have any data on regimen type and/or duration? If so, given this established increased risk of hyperglycemia among people living with HIV with longer exposure to ART and on certain regimens, authors should present median ART duration and ART regimen class in Table 1. If it is found that ART duration and/or class has a statistically significant relationship with hyperglycemia, authors should adjust for these variables in the multivariate analyses.

Response: Dear Dr. Emma M Kileel, I want to express my gratitude for your time, suggestions, and advice. Glucose levels considered for analysis were at the entry into HIV care, as recommended by National guidelines (Before ART initiation). Every patient has begun the ART regimen at some point (some faster, some later). The median ART duration was 3.58 (2.86). We did not adjust variables in the multivariate analysis because all our glucose measurements were taken before the ART regimen. Information about ART and mortality is not in the tables either because every patient is on ART, and we would not be able to find a statistically significant difference.

Comment: Authors state a major limitation of the paper is the potential underestimation of hyperglycemia because all glucose measurements with unknown fasting information were considered postprandial. In order to give the reader a sense of how much this may have impacted your results, authors should report what % of glucose measurements had unknown fasting information.

Response: Based on the given data, information about fasting glucose was unknown in 18.6% of cases. This information will be included in the paper as it was suggested. Thank you for your comment and advice.

Comment: Per journal requirement, an ethics statement needs to be included in the methods section of the manuscript.

Response: I added the following information in the method section: The study was approved by the Institutional Review Board of the Infectious Diseases, AIDS and Clinical Immunology Research Center (OHRP #: IRB00006106). All patients initiating HIV care provide informed consent on the use of information collected as part of routine clinical care for research. The study used secondary data extracted from the national HIV database as an anonymous dataset with no personal identities. Therefore, informed consent was waived by IRB.

Comment: Per journal requirement, authors need to make all raw data available or provide an explanation to data restrictions.

Response: Thank you very much for pointing it out. All raw data is uploaded on protocols.io

URL: <https://www.protocols.io/private/F851E67044A111EDB19C0A58A9FEAC02>

DOI: dx.doi.org/10.17504/protocols.io.3byl4jke8lo5/v1

Thank you very much for your help.

**Reviewer #2:** Overall: This is an interesting paper about the prevalence of hyperglycaemia and its impact on people living with HIV. It well-written paper, however, there are some problems with definition of hyperglycemia that need addressing. It highlights the importance of screening for dysglycaemia in individual living with HIV at the entry into care and as a part continually assessment.

Revision
1. Major changes

Comment: Line 90-92: Authors should justify why impaired fasting glucose levels were used to define hyperglycaemia. According to the guidelines, the postprandial glucose level > 7.8 mmol/L was based on an oral glucose tolerance test (OGTT) which is not highlighted in this paper.

Response: According to IDF (International Diabetes Federation), postprandial and postmeal plasma glucose levels should not rise above 7.8 mmol/l in healthy people. I am attaching the link below <https://www.in.gov/health/files/Guideline_PMG_final.pdf> ( page 7). Anything above 7.8 mmol/l is not considered as a normal range. I mentioned postmeal plasma glucose level and IDF in the text, as it was suggested. Thank you very much for your comment and help in clarifying the article.

Comment: The use of single point random glucose level may cause a misclassification of some patients as hyperglycaemic. A single high glucose level is less definitive (compared to OGTT or HbA1c) to classify dysglycemia. Consider highlighting this as a limitation of the study.

Response: The following statement, as added in recommendations **“**The use of single point random glucose level may cause misclassification of some patients as hyperglycaemic. To classify dysglycemia, a single high glucose level is less definitive (compared to OGTT or HbA1c). Unfortunately, OGGT or HbA1c has not been checked at the entry into HIV care; that’s why we did not have information about these variables. **“** Thank you very much for your thoughtful comments.

Comment: The authors should clarify if the glucose level considered for analysis were at the entry into HIV care as recommended by National guidelines or at any point during follow-up (or before ART initiation). Because both HIV and ART are implicated in development of hyperglycaemia/diabetes.

Response: Glucose levels considered for analysis were at the entry into HIV care, as recommended by National guidelines (Before ART initiation). Information was added in the article as suggested. Thank you for your advice and suggestion.

2. Minor changes

Abstract:

Comment: 1. Line 37: Please change 242 to Two hundred and forty-two

Response: Line 37: 242 was changed to Two hundred and forty-two. Thanks for the suggestion.

Comment:2. Line 38: Also, change 301 to Three hundred one

Response: Line 38: 301 was changed to Three hundred one. Thanks for the suggestion.

Introduction
Comment: Line 56: Change Type to type 2 diabetes

Response: Line 56: Type was changed to type 2 diabetes

Comment: Line 59: Please change Efavirenz to efavirenz.

Response: Line 59: Efavirenz was changed to efavirenz.

Comment: Line 59: Write PI, NRTI, and NNRT in full and abbreviate thereafter.

Response: Line 59: PI, NRTI, and NNRT were changed into protease Inhibitors (PI), nucleoside Reverse Transcriptase Inhibitors (NRTI), and some non-Nucleoside Reverse Transcriptase Inhibitors (NNRTI-s). Thank you very much for your suggestions.

Method
Comment: Line 81: What is the percentage of patients that were excluded from the analysis due to loss of follow-up?

Response: Line 81: 112 people (3.7%) were excluded from the analysis. I added it in methods, where I mentioned loss to follow-up. Thank you very much.

Comment: Line 93: rewrite ifit as if it

Response: Line 93: ifit was changed into if it.

Comment: Authors should add the under a method that the approval was obtained from the Ethics Committee of the Infectious Disease, AIDS, & Clinical Immunology Research Center.

Response: For more clarification, I added the following information: The study was approved by the Institutional Review Board of the Infectious Diseases, AIDS and Clinical Immunology Research Center (OHRP #: IRB00006106). All patients initiating HIV care provide informed consent on the use of information collected as part of routine clinical care for research. The study used secondary data extracted from the national HIV database as an anonymous dataset with no personal identities. Therefore, informed consent was waived by IRB.

Results
Comment: Line 120-121 (table 1):
• Please update the percentages for the population (total cohort) with hyperglycaemia to 8.3% and adjust accordingly throughout the manuscript including the abstract.
• Age category (30-39)- change 5.3 to 5.4%
• Age category (65+) – change to 25.8 %

Response: Table 1 has been updated. We decided to recheck our data once again to modify it. Thank you very much for your time and advice.

**Discussion**

Comment: Line 178-179: The authors should explain the possible reason why they found a lower prevalence of hyperglycaemia compared to other published studies.

Response: Thank you very much for pointing it out. I added an explanation in the text. The median age of the study group was 36 (IQR: 28-45) years old, so young participants could theoretically reduce the prevalence of hyperglycemia in the overall cohort. The prevalence of hyperglycemia was significantly higher in older people, which is not surprising because hyperglycemia and diabetes mellitus are more common in the older population, and the prevalence increases with age. Many other studies have found a similar relationship between increasing age and the prevalence of T2D among people living with HIV.

Comment: Line 190: Consider rephrasing ‘our study cannot be clearly explained’ statement.

Response: It was rephrased as suggested. Thank you for the suggestion.

Comment: Line 192-193: ‘The higher prevalence of hyperlycemia in people with advanced HIV should be interpreted considering previous findings indicating an association between low CD4 count and non-AIDS event’. It sounds there missing phrase? Consider rephrasing it.

Response: The sentence was rephrased: The higher prevalence of hyperglycemia in people with advanced HIV should be interpreted in light of previous findings linking a low CD4 cell count to non-AIDS events. Thank you for pointing it out. More information was added in front to clarify the sentence.

Comment: Under discussion, authors should add the mechanism linking HIV to hyperglycemia.

Response: Thank you for mentioning it. I added the mechanism linking HIV to hyperglycemia in the discussion.

Comment: Other factors such as obesity/ high BMI, and lifestyle that may contribute to hyperglycaemia/impaired fasting glucose were not assessed in this study. Should be highlighted as a limitation.

Response: Sentence: “Other factors such as obesity/ high BMI, and lifestyle that may contribute to hyperglycemia/impaired fasting glucose were not assessed in this study” was added in limitations. Thank you very much for your advice.

General comment:
Authors should leave the space between the last word and reference consistent throughout the manuscript.
Modify the font type for references

Response: It was fixed as suggested. Thank you very much for your advice.

Reviewer #1: **Yes:** Emma M. Kileel

Reviewer #2: No

Editor

1.Please ensure that your manuscript meets PLOS ONE's style requirements, including those for file naming. The PLOS ONE style templates can be found at

https://journals.plos.org/plosone/s/file?id=wjVg/PLOSOne_formatting_sample_main_body.pdf and

<https://journals.plos.org/plosone/s/file?id=ba62/PLOSOne_formatting_sample_title_authors_affiliations.pdf>.

Response: Thank you very much. It was formatted based on links.

2. Please provide additional details regarding participant consent. In the ethics statement in the Methods and online submission information, please ensure that you have specified (1) whether consent was informed and (2) what type you obtained (for instance, written or verbal, and if verbal, how it was documented and witnessed). If your study included minors, state whether you obtained consent from parents or guardians. If the need for consent was waived by the ethics committee, please include this information.

If you are reporting a retrospective study of medical records or archived samples, please ensure that you have discussed whether all data were fully anonymized before you accessed them and/or whether the IRB or ethics committee waived the requirement for informed consent. If patients provided informed written consent to have data from their medical records used in research, please include this information.

Thank you for your thoughtful comment. It was added in method section.

3. We note that the grant information you provided in the ‘Funding Information’ and ‘Financial Disclosure’ sections do not match. When you resubmit, please ensure that you provide the correct grant numbers for the awards you received for your study in the ‘Funding Information’ section.

Response: Our research did not have any grants. The author(s) received no specific funding for this work.

4. Thank you for stating the following in the Acknowledgments Section of your manuscript:

“Many thanks to all the doctors and staff of the Infectious Diseases, AIDS & Clinical Immunology Research Center who support the implementation of the HIV/AIDS Program”

We note that you have provided funding information that is not currently declared in your Funding Statement. However, funding information should not appear in the Acknowledgments section or other areas of your manuscript. We will only publish funding information present in the Funding Statement section of the online submission form.

Please remove any funding-related text from the manuscript and let us know how you would like to update your Funding Statement. Currently, your Funding Statement reads as follows:

“The study has been conducted with internal resources of Infectious Diseases, AIDS & Clinical Immunology Research Center”

Please include your amended statements within your cover letter; we will change the online submission form on your behalf.

Response: The author(s) received no specific funding for this work. was added in the cover letter. Information in acknowledgements was updated. Thank you for your thoughtful comments, suggestions and advice.

5. Thank you for stating the following in your Competing Interests section:

“The authors have no conflicts of interest to disclose.”

Please complete your Competing Interests on the online submission form to state any Competing Interests. If you have no competing interests, please state "The authors have declared that no competing interests exist."", as detailed online in our guide for authors at http://journals.plos.org/plosone/s/submit-now

This information should be included in your cover letter; we will change the online submission form on your behalf.

Response: ‘’The authors have declared that no competing interests exist’’ was added in the cover letter. Thank you for your advice.

6. In your Data Availability statement, you have not specified where the minimal data set underlying the results described in your manuscript can be found. PLOS defines a study's minimal data set as the underlying data used to reach the conclusions drawn in the manuscript and any additional data required to replicate the reported study findings in their entirety. All PLOS journals require that the minimal data set be made fully available. For more information about our data policy, please see <http://journals.plos.org/plosone/s/data-availability>.

Upon re-submitting your revised manuscript, please upload your study’s minimal underlying data set as either Supporting Information files or to a stable, public repository and include the relevant URLs, DOIs, or accession numbers within your revised cover letter. For a list of acceptable repositories, please see http://journals.plos.org/plosone/s/data-availability#loc-recommended-repositories. Any potentially identifying patient information must be fully anonymized.

Important: If there are ethical or legal restrictions to sharing your data publicly, please explain these restrictions in detail. Please see our guidelines for more information on what we consider unacceptable restrictions to publicly sharing data: http://journals.plos.org/plosone/s/data-availability#loc-unacceptable-data-access-restrictions. Note that it is not acceptable for the authors to be the sole named individuals responsible for ensuring data access.

We will update your Data Availability statement to reflect the information you provide in your cover letter.

Response : Thank you very much for the important information. Here are URL and DOI from protocols.io

URL: <https://www.protocols.io/private/F851E67044A111EDB19C0A58A9FEAC02>

DOI: dx.doi.org/10.17504/protocols.io.3byl4jke8lo5/v1

7. Please include your full ethics statement in the ‘Methods’ section of your manuscript file. In your statement, please include the full name of the IRB or ethics committee who approved or waived your study, as well as whether or not you obtained informed written or verbal consent. If consent was waived for your study, please include this information in your statement as well.

Response: Full Ethics Statement was added in the methods section. thank you for your help.

Please review your reference list to ensure that it is complete and correct. If you have cited papers that have been retracted, please include the rationale for doing so in the manuscript text, or remove these references and replace them with relevant current references. Any changes to the reference list should be mentioned in the rebuttal letter that accompanies your revised manuscript. If you need to cite a retracted article, indicate the article’s retracted status in the References list and also include a citation and full reference for the retraction notice.

Response: References were double-checked. Thank you for your suggestions.
